# Effects of Repetitive Upsetting Extrusion on the Microstructure and Texture of GWZK124 Alloy under Different Starting Temperatures

**DOI:** 10.3390/ma12152437

**Published:** 2019-07-31

**Authors:** Guanshi Zhang, Zhimin Zhang, Yingze Meng, Zhaoming Yan, Xin Che, Xubin Li

**Affiliations:** 1 School of Materials Science and Engineering, North University of China, Taiyuan 030051, China; 2 College of Mechatronics Engineering, North University of China, Taiyuan 030051, China

**Keywords:** repetitive upsetting extrusion, Mg-Gd-Y-Zn-Zr, microstructure, dynamic recrystallization, texture

## Abstract

The effects of repetitive upsetting extrusion under different starting temperatures on the microstructure and texture of GWZK124 alloy were investigated. The results clearly showed that the particles and second phases induced dynamic recrystallization (DRX), which can be explained by the particle-stimulated nucleation (PSN) mechanism. It was shown that grain refinement during repetitive upsetting extrusion (RUE) is dominated by a complicated combination of continuous dynamic recrystallization and discontinuous dynamic recrystallization. The RUEed alloys under different starting temperatures exhibited a bimodal microstructure comprising fine DRXed grains with weak texture and coarse deformed grains with strong texture. The DRXed grains could weaken the texture. As the RUE starting temperature decreased, the average grain size increased and the volume fraction of DRXed grains decreased.

## 1. Introduction

Magnesium (Mg) alloys have been applied in various structural components in the aerospace, automotive and aircraft industries owing to their good damping capacity, low density, excellent creep resistance and so on [1,2,3]. Compared to other conventional structural alloys, Mg alloy has poor strength and inferior ductility at room temperature (RT) due to its hexagonal close-packed (hcp) lattice structure [4,5,6,7]. Grain refinement is one obvious way to simultaneously improve its strength and ductility. Repetitive upsetting extrusion (RUE), a kind of severe plastic deformation (SPD) technique, is attractive for its potential to fabricate high volumes of blanks [8], which has significant application in industry. Furthermore, it is highly adaptable for refining grains of hard-to-deform metal such as Mg alloy because it enables three-dimensional compression stresses to be exerted during deformation, while avoiding sample cracking [9]. Previous researches on materials such as LY12 [10], AZ61 [11] and AZ91D [12], have revealed that RUE processing can refine grains and the second phases.

In recent years, many researchers have been working hard to develop novel alloy compositions to improve the strength and ductility of Mg alloy, and to achieve light weight applications. Rare earth (RE) bearing Mg alloys are noted for their excellent properties at high temperature and RT due to the formation of heat-resistant precipitates [13]. Among kinds of RE Mg alloys, Mg-RE-Zn alloys have been the focus of numerous researchers owing to their reasonable ductility and superior high strength [14]. It is believed that the new and unique long-period stacking ordered (LPSO) phases in Mg-Gd-Y-Zn-Zr alloys contribute to improving both their ductility and strength [15,16]. Moreover, the kink mechanism of intragranular LPSO phases can accommodate strain, which can also contribute to the excellent properties of the Mg-Gd-Y-Zn-Zr alloys [17,18,19,20]. Recently, the ultimate tensile strength (UTS) of Mg-1.8Gd-1.8Y-0.7Zn-0.2Zr (at %) alloy reached 542 MPa, subsequent to its preparation by hot extrusion followed by aging treatment [21]. During the aging treatment, the fine and dense precipitates appeared around the grain boundaries of the dynamically recrystallized grains, which was the main reason for its excellent properties. Xu et al. produced Mg-8.2Gd-3.8Y-1.0Zn-0.4Zr (wt %) alloys by hot rolling and aging treatment, resulting in the UTS increasing to 517 MPa and the fracture elongation increasing to 4.5% [22].

The aim of this article is to demonstrate the effects of RUE on the microstructure and texture of GWZK124 alloy under different starting temperatures. In addition, the grain refinement mechanism of RUE on the as-homogenized GWZK124 alloy was investigated.

## 2. Materials and Methods

**Figure 1:**
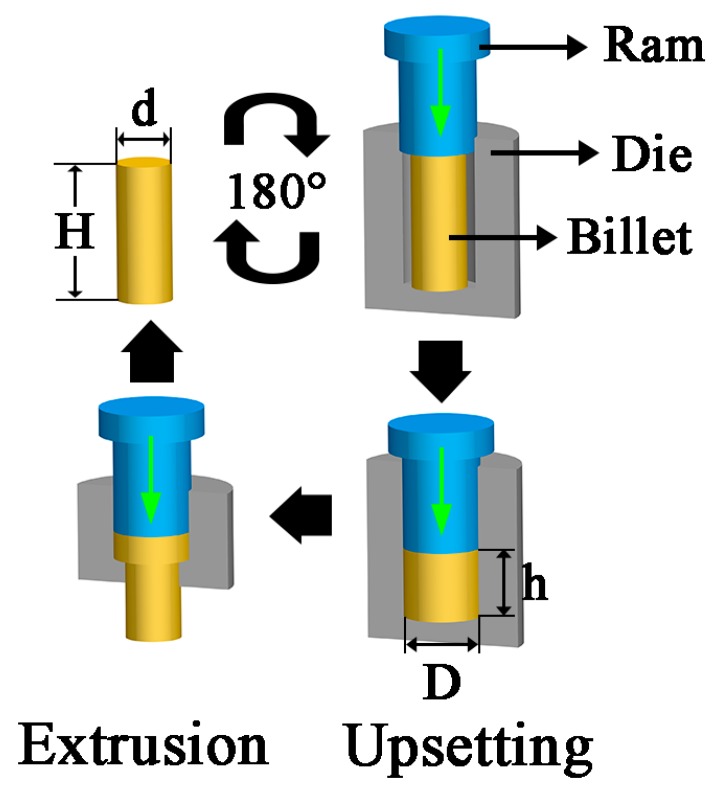
The principle of the repetitive upsetting extrusion (RUE) processing.

The composition of the studied alloy was Mg-12.1Gd-4.0Y-2.2Zn-0.34Zr (wt %), which is denoted here as GWZK124. A cylindrical ingot measuring 3000 mm in length and 330 mm in diameter was prepared by the semi-continuous casting method. The positions of all of the samples were at the center of the ingot, and these cylinders were φ20 mm × 50 mm. Subsequent homogenization was performed at a temperature of 520 °C for 16 h and then quenched in water at a temperature of 70 °C to obtain uniformity in the initial microstructure. The schematic of the RUE is presented in Figure 1 [10]. The RUE processing combined upsetting and extrusion and the two processes were alternated in each pass, which allowed for the accumulative strain. The RUE dies were preheated to the given temperature in the resistance furnace, and then reserved for 30 min before RUE processing. The as-homogenized samples were preheated at the given temperature for 10 min before RUE processing to homogenize the temperature of the samples. To achieve relatively homogeneous deformation, a graphite-based mixture was used as the lubricant.

**Figure 2:**
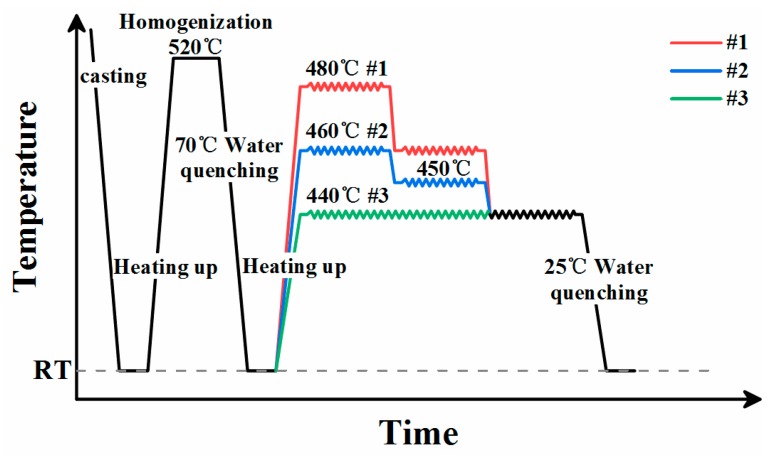
The schematic illustrations of experimental procedures for sample #1, #2 and #3.

These as-homogenized samples were RUEed on an Instron 3382 tensile machine equipped with a resistance furnace at a ram speed of 4mm/min and they were divided into 3 groups. The first group was RUEed under decreasing temperatures from 480 °C to 440 °C with a drop of 20 °C every one RUE pass (defined as sample #1). The second group was RUEed under decreasing temperatures from 460 °C to 440 °C with a drop of 10 °C every one RUE pass (defined as sample #2). The third group was RUEed at 440 °C (defined as sample #3). The schematic illustration of the experimental procedures for sample #1, #2 and #3 are shown in Figure 2. The temperature of the resistance furnace was monitored using a K-type thermocouple. The magnitude of the accumulative strain used the following formula: ∑Δε = 4n × ln (D/d) (where n is the number of passes through the upsetting and extrusion dies) [23]. In this research, D and d were 28 mm and 20 mm, respectively. RUE was carried out up to a maximum accumulative strain of ∑Δε = 4.04, i.e., a total of three passes. All samples were quenched immediately in the water at RT after deformation.

For the phase analysis, X-ray diffraction (XRD) was performed on the SmartLab, in the 2θ diffraction angle from 20° to 90°. To evaluate the homogeneity of the RUEed samples, the edge and the center of the samples along the longitudinal section were observed. The evolved microstructures were prepared for optical microscopy (OM), scanning electron microscopy (SEM, Hitachi SU5000, Tokyo, Japan) and electron backscatter diffraction (EBSD, EDAX Inc., Mahwah, NJ, USA). OM observation was carried out using the Zeiss Axio Imager A2m (Zeiss, Oberkochen, Germany). To evaluate the grain size, the samples were etched in a solution of 3 mL distilled water, 2 g picric acid, 2 mL acetic acid and 20 mL ethanol. The average size and volume fractions of grains were simply measured through Image-Pro Plus 6.0 software based on OM images. EBSD collection was carried out using a Hitachi SU5000 SEM equipped with an EDAX-TSL EBSD system. The EBSD data were analyzed using orientation imaging microscopy (OIM) software version 7.3 to obtain the texture and other information about the samples. DRXed grains were defined as grains with an average grain size of ≤10 μm.

## 3. Results and Discussion

### 3.1. Microstructures of the As-Cast and As-Homogenized GWZK124 Alloys

**Figure 3:**
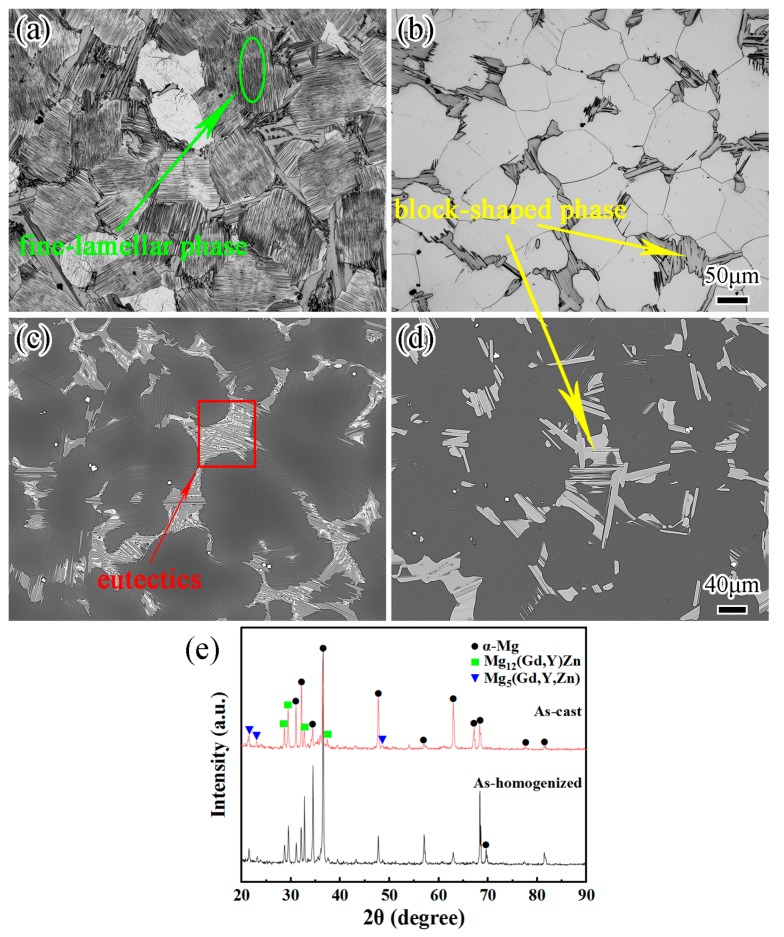
Optical microscopy (OM) micrographs of the GWZK124 alloys: (**a**) as-cast; (**b**) as-homogenized, and their corresponding back-scattered electron (BSE) micrographs (**c**,**d**). XRD patterns of GWZK124 alloys in as-cast and as-homogenized states (**e**).

The OM and back-scattered electron (BSE) micrographs of the as-cast GWZK124 alloy are shown in Figure 3a,c. The as-cast alloy consisted of α-Mg matrix and network eutectics. Some oriented fine-lamellar phases could be seen inside the grains, and these intragranular fine-lamellar phases were considered as the 14H-LPSO phases [24]. XRD patterns demonstrated the existence of α-Mg matrix, Mg_12_(Gd,Y)Zn and Mg_5_(Gd,Y,Zn) phases in the as-cast alloy (Figure 3e). After homogenization at 520 °C for 16 h (Figure 3b,d), the alloy consisted of α-Mg matrix with a grain size of 100 μm and interdendritic block-shaped phases. In comparison with the as-cast alloy, there was no obvious grain growth for the as-homogenized alloy owing to the pinning effect produced by the block-shaped phases at the triple junctions. The network of grain boundary eutectics and fine-lamellar phases disappeared because of the dissolution of Gd, Y and Zn elements. Many block-shaped phases remained, which indicated that the block-shaped phases had higher thermal stability. It could be verified that Mg_5_(Gd,Y,Zn) belonged to the network phase and Mg_12_(Gd,Y)Zn belonged to the block-shaped phase (Figure 3a,b). Previously, it has been found that the Mg_12_(Gd,Y)Zn was the 14H-LPSO phase [25]. It could be seen that the α-Mg matrix occupied the dark contrast areas, and the grey block-shaped phases were distributed around the grain boundary (Figure 3d).

### 3.2. Microstructures of the RUEed Alloys under Different Starting Temperatures

**Figure 4:**
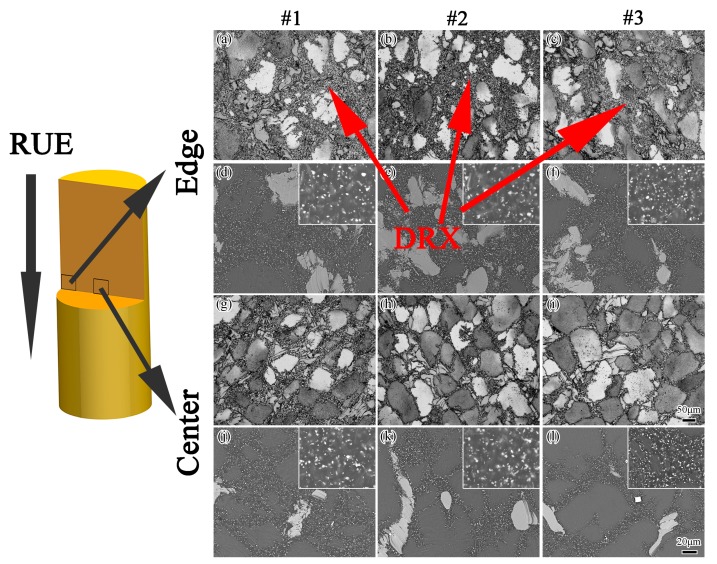
OM (**a**–**c**,**g**–**i**) and corresponding BSE (**d**–**f**,**j**–**l**) micrographs of the edge and the center of the samples RUEed under different starting temperatures. The magnified images of the particle phases are shown in the upper right corner.

Figure 4 shows the OM and corresponding BSE images of the edge and the center of the samples along the RUE direction. It can be easily observed that the microstructure of the samples exhibited a special bimodal distribution in which deformed grains distributed along the RUE direction and small grains had an average grain size of about 3.8 μm. Many oriented fine-lamellar phases were formed inside the grains, and some particle phases were found at the triple points of DRXed grain boundaries, indicating that dynamic precipitation happened during RUE processing. These fine-lamellar phases and particle phases were the 14H-LPSO and Mg_5_(Gd,Y,Zn), respectively [26]. Meanwhile, it could also be seen that some interdendritic block-shaped phases were broken up and heterogeneously distributed around the grain boundary. The dynamic recrystallization (DRX) was believed to occur around the grain boundaries of the deformed grains and broken second phases, where dislocations were accumulated during deformation. It is widely known that the particles and second phases induce DRX, which can be explained by the particle-stimulated nucleation (PSN) mechanism [27]. Because of the inhibition of dislocation displacement, the dislocations piled up between the block-shaped phases and matrix. It is supposed that the PSN involved a rapid sub-grain boundaries migration in the areas around the block-shaped phases during RUE processing. The sub-grain boundaries absorbed the piling up dislocations during migration, increased the misorientation and gradually formed high-angle grain boundaries (HAGBs), thus realizing the DRX process. The grain measurement showed that the average grain size decreased to about 45 ± 5 μm in the center of the sample #1 and to about 27 ± 3 μm at the edge (Figure 4a,g).

Under different starting temperatures, the grain size distribution was still heterogeneous with fine grains along the grain boundaries, and some initial coarse grains (Figure 4). It could also be seen that lower starting temperatures enhanced the precipitation of the particle phases (Figure 4d,f). When the RUE starting temperatures were decreased, there was insufficient stored energy in the alloy, thus the degree of DRX was decreased. Therefore, the deformation temperature played an important role in the formation of DRX. The deformation temperature had an effect on the dislocation density, dislocation migration and the formation of sub-grain structures [28]. The DRXed grain size did not show much difference despite the existence of different volume fractions of DRXed grains. The non-uniformity of deformation with different starting temperatures was observed, for example, the sample from the center of the longitudinal section (Figure 4h,i) exhibited a coarser structure than that taken from the edge (Figure 4b,c). As the RUE starting temperature decreased, the grain size gradually increased.

### 3.3. Microtexture Evolution of the RUEed Alloys

**Figure 5:**
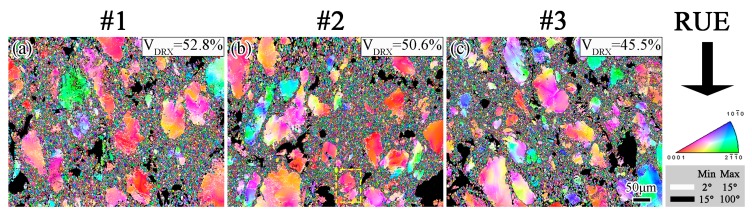
OIM maps of the edge of sample #1 (**a**), #2 (**b**) and #3 (**c**). The color-code of the stereographic triangle shows the crystallographic orientation of hcp crystal. Black lines indicate HAGBs larger than 15° and white lines indicate low-angle grain boundaries (LAGBs) between 2° and 15°.

For the purpose of discussing the mechanism of DRX and texture evolution, the RUEed alloys were investigated using EBSD. Figure 5 exhibits the OIM maps close to the edge of the alloys RUEed under different starting temperatures, however, only α-Mg could be analyzed and the black areas or irregular aggregation of dots represent the second phases with low confidence index values because the OIM system could not analyze their Kikuchi diffraction patterns [17]. Black lines indicate HAGBs larger than 15° and white lines indicate low-angle grain boundaries (LAGBs) between 2 °C and 15°.

The deformation bands and twins were commonly observed in the Mg-Gd-Y alloys [29] and Mg–Al-Zn alloys [30,31,32] deformed under other deformation conditions. However, they could not be seen in the RUEed alloys in this study. Hence, grain refinement was mainly caused by DRX. The DRX mechanism in Mg alloys could be classified into two groups: continuous dynamic recrystallization (CDRX) and discontinuous dynamic recrystallization (DDRX). In accordance with the OM observations that all the alloys RUEed under different starting temperatures exhibited the typical bimodal microstructure, the fine DRXed grains showed almost random crystallographic orientation, and an evident color gradient could be seen in many large grains, which suggested that sub-grain boundaries and dense dislocations in the large grains induced the occurrence of misorientation.

**Figure 6:**
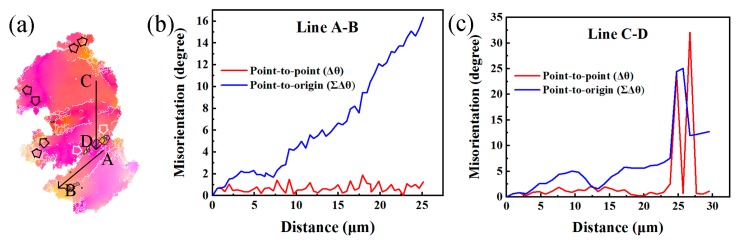
(**a**) Extracted grains from the edge of sample #2 (highlighted by yellow dashed boxes in Figure 5b), and (**b**,**c**) show the typical point-to-point and point-to-origin misorientations along lines AB and CD marked in (**a**).

Figure 6b,c show the point-to-point misorientations, Δθ, and point-to-origin misorientations, ∑Δθ, along the arrows AB and CD. The misorientation increased gradually up to 16 owing to the lattice rotation relative to the origin point. However, some DRXed grains also formed along the LABs, marked by the white arrows (Figure 6a). Usually, LAGBs result from the accumulation of dislocations and formation of sub-GBs in the large grains [33,34]. With the increasing strain, these LAGBs trapped more mobile dislocations and transformed themselves into HAGBs, ultimately turning sub-grains into DRXed grains, which indicated the operation of CDRX. DRX relied on the morphologies of the second phases. It was verified that large second phases enhance the accumulation of strain gradient during deformation, which is necessary for the PSN mechanism to operate [27]. In addition, as indicated by the black arrows, bulges could be observed along the initial coarse boundary. The size of the bulges was equal to that of the DRXed grains, which indicates that DDRX also took place during RUE processing.

**Figure 7:**
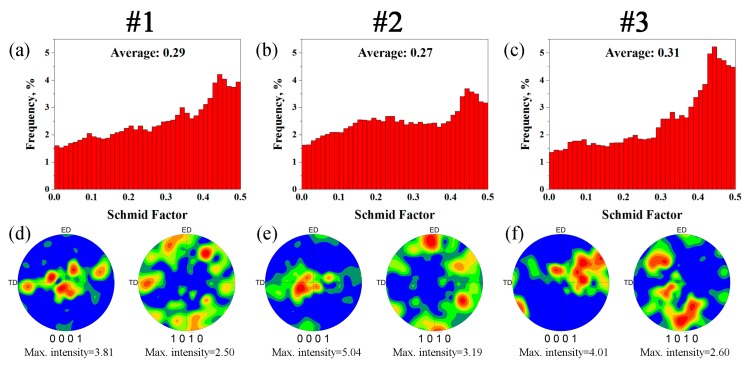
(0001) <11–20> Schmid factor distribution histograms (**a**–**c**) and pole figures (**d**–**f**) of the edge of sample #1 (**a**,**d**), #2 (**b**,**e**) and #3 (**c**,**f**).

**Figure 8:**
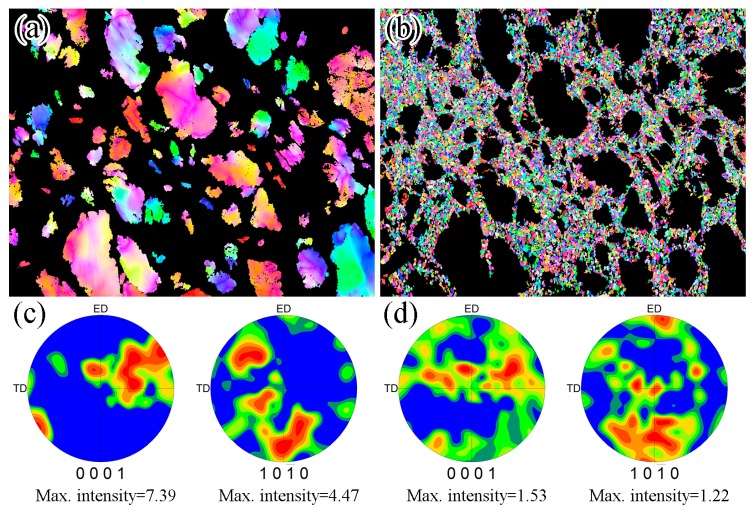
OIM maps and corresponding EBSD-derived pole figures of the edge of the sample #3: (**a**,**c**) deformed grains; (**b**,**d**) DRXed grains.

Figure 7a–c present the (0001) <11–20> Schmid factor distribution maps of the edge of sample #1, #2 and #3. The peak value of the (0001) <11–20> Schmid factor was mainly distributed around 0.45. Texture evaluation of the RUEed alloys under different starting temperatures, the (0001) and (10–10) pole figures are presented in Figure 7d–f. It was found that all the alloys presented the (0001) planes parallel to the RUE direction, which is the typical extrusion texture of Mg alloy. The effects of deformed and DRXed grains on the texture of the alloys RUEed under different starting temperatures were similar. The OIM maps and corresponding pole figures in the deformed and DRXed grains of sample #3 are partitioned (Figure 8). The deformed grains had a stronger basal texture (Figure 8c), but the (0001) pole figures distribution of the DRXed grains was rather dispersive (Figure 8d). The maximum basal texture intensity of DRXed grains was only 1.5, and the texture randomization associated with PSN of recrystallization has been reported in the Al alloys [35] and Mg alloys [36]. Therefore, the high volume fraction of DRXed grains could weaken the texture. Such DRXed grains could shorten the slip distance of the dislocations, which led to the release of stress concentration at the grain boundaries, and enhanced the ductility of the alloys. The bimodal microstructure had a similar effect on the mechanical properties of the extruded Mg-Zn-Y alloys [37]: The deformed grains showed strong texture, which together with the LPSO phases, can strengthen the alloys. However, the DRXed grains exhibited weak texture, which can enhance the ductility.

## 4. Conclusions

1. The as-cast alloy consisted of α-Mg, fine-lamellar phases and eutectics. After homogenization, there was no obvious grain growth owing to the pinning effect produced by the block-shaped phases. Mg_5_(Gd,Y,Zn) belonged to network eutectics and Mg_12_(Gd,Y)Zn belonged to block-shaped phases.
2. The oriented fine-lamellar phases and particle phases dynamically precipitated during repetitive upsetting extrusion (RUE). The particles and second phases induced dynamic recrystallization (DRX), which can be explained by the particle-stimulated nucleation (PSN) mechanism. The samples from the center of the longitudinal section exhibited a coarser structure than those taken from the edge.
3. The RUEed alloys under different starting temperatures exhibited the typical bimodal distribution; the deformed grains showed strong texture and the DRXed grains showed weak texture. The grain refinement mechanisms of RUEed alloys were caused by continuous dynamic recrystallization (CDRX) and discontinuous dynamic recrystallization (DDRX). The DRXed grains could weaken the texture. With a decrease in the RUE starting temperatures, the average grain size and the precipitation of particle phases increased and the volume fraction of DRXed grains decreased.
